# Environmental epigenetics in zebrafish

**DOI:** 10.1186/s13072-017-0154-0

**Published:** 2017-10-05

**Authors:** Vincenzo Cavalieri, Giovanni Spinelli

**Affiliations:** 1 Laboratory of Molecular Biology and Functional Genomics, Department of Biological, Chemical and Pharmaceutical Sciences and Technologies (STEBICEF), University of Palermo, Viale delle Scienze Edificio 16, 90128 Palermo, Italy; 2 Zebrafish Laboratory, Advanced Technologies Network (ATeN) Center, University of Palermo, Viale delle Scienze Edificio 18, 90128 Palermo, Italy

**Keywords:** Environmental epigenetics, Zebrafish, DNA methylation, Methylome, Histone modifications, Embryogenesis, Toxicant, Transgenerational inheritance

## Abstract

It is widely accepted that the epigenome can act as the link between environmental cues, both external and internal, to the organism and phenotype by converting the environmental stimuli to phenotypic responses through changes in gene transcription outcomes. Environmental stress endured by individual organisms can also enforce epigenetic variations in offspring that had never experienced it directly, which is termed transgenerational inheritance. To date, research in the environmental epigenetics discipline has used a wide range of both model and non-model organisms to elucidate the various epigenetic mechanisms underlying the adaptive response to environmental stimuli. In this review, we discuss the advantages of the zebrafish model for studying how environmental toxicant exposures affect the regulation of epigenetic processes, especially DNA methylation, which is the best-studied epigenetic mechanism. We include several very recent studies describing the state-of-the-art knowledge on this topic in zebrafish, together with key concepts in the function of DNA methylation during vertebrate embryogenesis.

## Background

Research in the field of environmental epigenetics focuses on how gene regulatory mechanisms operate on chromatin, in the absence of changes in the genome sequence, during adaptive responses to external stimuli [1–3]. The main epigenetic mechanisms include DNA methylation, histone post-translational modifications, and replacement of canonical histones by specialized histone variants, nucleosome density, three-dimensional chromatin organization, noncoding RNAs, and transcription factor regulatory networks [4–11]. Close interlinking among all these mechanisms establishes the so-called epigenotype displayed by a given cell/organism within a given environment. The dynamic nature of such a finely tuned epigenetic equilibrium implies that the epigenotype fluctuates rather rapidly in response to external stimuli, potentially allowing gradual adaption of genome transcriptional outputs and phenotype variation [12, 13]. On the other hand, especially in the case of the germline and stem cells, some particular epigenetic patterns may persist in the chromatin across generations, constituting the basis for long-term adaption [14, 15].

A growing body of evidence shows that there are critical time windows during embryogenesis and primordial germ cells specification in which the epigenome is extremely sensitive to environmental cues, which can therefore modify the epigenetic information both within developing individuals and across generations [16, 17].

Because of the variability in reproductive and developmental processes, and their response to environmental stress, a wide variety of model and non-model organisms have been employed to study both the individual and transgenerational inheritance of epigenetic information [18–22].

The focal point of this review is the use of zebrafish to evaluate DNA CpG methylation, which is the best-studied epigenetic mechanism among those that can covalently modify DNA. It consists in the enzymatic transfer of a methyl group from the *S*-adenosyl-methionine donor to the 5th carbon position of a cytosine pyrimidine ring [23]. Accumulation of 5-methyl cytosines mostly occurs in the so-called CpG islands, which are genomic regions with densely clustered CG dinucleotides. Control of gene expression can be affected by context-dependent changes in CpG methylation. In particular, while CpG methylation at promoters generally causes stable transcriptional gene silencing [4], high levels of CpG methylation within the gene body are associated with highly expressed genes [24]. CpG methylation also plays fundamental roles in genomic imprinting [25] and X-chromosome inactivation [26].

Various organisms contain cytosine methylation in CpA, CpT, and CpC dinucleotides, which are collectively referred to as non-CpG methylation [27–29]. The overwhelming majority of non-CpG methylated sites, primarily CpA dinucleotides, are enriched in brain tissue and pluripotent cells compared to other differentiated cell types [24, 28, 29]. However, the functional significance of this occurrence is poorly understood in the vertebrate genome, and it will not be covered in this review.

DNA methylation patterns are primarily imposed by de novo DNA methyltransferases (Dnmts), and then, they are semi-conservatively transferred onto the newly synthesized DNA strand after each cell division by a maintenance Dnmt [23, 30, 31]. Reversion of DNA methylation, especially during embryogenesis, is thought to be obtained by a passive replication-dependent mechanism involving the inhibition of Dnmts [32–34]. Alternatively, a multistep process embracing both ten-eleven translocation (Tet) proteins and the DNA repair machinery mediates active demethylation [34–37]. Due to the combination of all of these events, DNA methylation patterns are highly dynamic throughout embryonic development, particularly during epigenetic reprogramming, in which the bulk of paternal and maternal epigenetic asymmetries become harmonized into the zygotic genome [38–42].

## The zebrafish model

Zebrafish (*Danio rerio*) are small tropical freshwater fish native to the inland water bodies of the Himalayan region [43]. These vertebrate organisms are small, the adults being about 2–3 cm in length, and can be maintained relatively cheaply in laboratory. They have a short life cycle and generation time, as well as high fecundity, single female individuals being capable of generating hundreds of eggs per week [44]. Altogether, these features allow rapidity and high statistical power for downstream experimental procedures.

The directly developing embryos grow outside the maternal fish, elaborating the general body plan and organ systems within 48 h from fertilization [45]. Furthermore, zebrafish embryos appear quite translucent to microscopic observation, facilitating noninvasive live imaging of morphogenetic processes at a single-cell level in the context of the whole organism [46, 47].

Importantly, zebrafish embryos are relatively permeable to water-soluble molecules, being ideally suited for drug discovery and monitoring of pollutants [48]. The typical experimental strategy consists of large-scale pharmacological/toxicological screenings based on exposure to chemical compounds followed by high-throughput molecular studies. As discussed below, the zebrafish has recently been proved to be a premier model to explore changes in the epigenetic state, especially DNA methylation, following exposure to several environmental stressors.

It is worth mentioning that it is estimated that the zebrafish genome has approximately 70% homology to human genes [49] and that ~ 99% of embryonic-essential fish genes are homologs in human embryonic development [50]. Last but not least, the main epigenetic mechanisms and events, especially those occurring during germ cell programming, are common to zebrafish and mice [51, 52]. Undoubtedly, these aspects are of fundamental importance in allowing researchers to extrapolate results to other vertebrates, including humans.

## DNA methylation in zebrafish

DNA methylation machinery and mechanisms in zebrafish are generally conserved with those of mammals [53–56], with the significant exception that fish do not require imprinting of genes or sex chromosomes for viability [30, 57]. This feature provides a simplified system for exploring methylome dynamics in response to environmental challenges during the so-called epigenetic reprogramming process, which includes the establishment of DNA methylation patterns during vertebrate embryogenesis [58].

Measurements of overall DNA methylation at different developmental time points during zebrafish development revealed that over 80% of CpGs are methylated, and modest gain from this high baseline occurs as the embryo progresses toward gastrulation [51, 52, 59–62]. Interestingly, the paternal DNA methylation pattern is maintained throughout early embryogenesis, while the hypomethylated maternal DNA is reprogrammed to a pattern similar to that of the sperm [51, 52]. Moreover, the overall DNA methylation level in zebrafish is higher than those of endothermic animals, probably due to a lower deamination rate of methylated cytosine to thymine [53, 63].

Zebrafish possess multiple *dnmt* genes representing the homologs of mammalian maintenance *dnmt1* [64], and de novo *dnmt3a* (*dnmt3a1* and *2*) and *dnmt3b* (*dnmt3b1*, *2*, *3*, and *4*), which arose following the genome duplication event characterizing the teleost fish lineage, as well as tandem gene duplications [65, 66]. Zebrafish also contain the three Tet family proteins shared in vertebrates, but fish embryos do not express them during early development [67]. Nonetheless, a recent study has shown that a massive wave of Tet-dependent DNA demethylation begins at about 24 h post-fertilization, temporally encompassing the so-called phylotypic stage [68], which is the period in which developing embryos of species in the same phylum display maximal similarity. Strikingly, such an epigenome reconfiguration is evolutionary conserved across zebrafish, Xenopus, and mouse [68], suggesting that DNA demethylation could be a preeminent epigenetic mechanism accounting for co-regulation of key developmental genes in multiple vertebrate species. Accordingly, the chromatin contexts exhibiting DNA hypomethylation contain thousands of enhancers embedded into gene regulatory networks controlling body plan and organ formation [68].

## Impact of environmental compounds on DNA methylation during zebrafish embryogenesis

Some recent studies have primarily focused on global DNA methylation changes and phenotypic alterations triggered by environmental pollutants during epigenetic reprogramming of zebrafish embryos. An overview of the studies examined in this review is shown in Table 1.

**Table 1:** Overview of studies examining the epigenetic effects in zebrafish embryos exposed to several compounds

Compound	Epigenetic effect	References
Benzo[*a*]pyrene	Global and gene-specific hypomethylation Upregulation of *dnmt3b2* Downregulation of *dnmt1* and *dnmt3a2* Stimulation of Gnmt activity	[62, 99]
Androgens	Global hypomethylation	[77]
Arsenic	Differential spatiality-specific global methylation	[84]
Estrogens	Gene-specific hypomethylation: *vasa*	[85]
Nickel, cadmium	Gene-specific hypermethylation: *vasa*	[85]
Bisphenol-A	Gene-specific alterations of DNA methylation Downregulation of *dnmt1*, *dnmt3b3*, *dnmt3b4* ^a^	[85, 122, 123]
Perfluorooctanoic acid	Gene-specific alterations of DNA methylation	[85]
S-(+) fipronil	Global and gene-specific hypermethylation	[90]
TCDD	Gene-specific hypomethylation: *cfos* Gene-Specific hypermethylation: *ahrra* Upregulation of *dnmt1* and *dnmt3b2* Downregulation of *dnmt3a1*, *dnmt3b1* and *dnmt3b4*	[85, 92, 93]
Lead	Overall DNA hypomethylation Inhibition of Dnmt1 activity Downregulation of *dnmt3b1* and *dnmt3b3*	[98]
Heat stress/copper	Upregulation of *dnmt3* genes	[103]
Methylmercury	Differential methylation of noncoding DNA	[92]
MEHP, 5-azacytidine	Upregulation of *dnmt1*, *dnmt3b1* and *dnmt3b2* Downregulation of *dnmt3a1* and *dnmt3a2* Overall hypomethylation^b^	[109]
Ethanol	Upregulation of specific miRNAs	[111]
Perfluorooctane sulfonate	Differential alterations of miRNAs abundance	[113]
DZNep	Gene-specific depletion of H3K27me3 and H3K9me3	[114]

^a^Transgenerational effect; ^b^ in F0 liver of female fish

One of these studies reported that the overall DNA methylation level of developing embryos continuously exposed to benzo[*a*]pyrene, a well-known carcinogen and epigenetic modifier [69–71], was about half of that of control untreated embryos [62]. Consistently, significant loss of methylation in the promoter region, as well as concomitant increase in mRNA transcription, was specifically detected for the *vasa* gene [62]. Because *vasa* is required for differentiation and migration of primordial germ cells [72–74], the authors claimed that irregular epigenetic modulation of *vasa* gene expression could in turn arouse reproductive toxicity.

In a more recent study, the established relationship between fetal androgen exposure and reproductive defects in animal models [75, 76] inspired exploration of global DNA methylation in ovaries of adult zebrafish antecedently exposed to testosterone or dihydrotestosterone during embryogenesis [77]. Interestingly, the authors observed a biphasic dose response in the methylome of androgenized zebrafish, with an inverse relationship between global methylation status and androgen dose exposure. This finding is in accordance with evidence reported by similar studies in other organisms [78, 79], and it has been explained by downregulation of androgen receptors at higher exposure levels or adaptive responses through complex signaling pathways.

Another pertinent example refers to exposure of zebrafish embryos to arsenic, an environmental contaminant known to have adverse effects on human health by causing a series of cancers and cardiovascular and neurological diseases [80–83]. Consistent with this, when used at a concentration of 2.0 mM, sodium arsenite inflicted severe malformations of neural and cardiac structures in developing zebrafish and provoked substantial changes in the genomic DNA methylation pattern throughout the embryonic body [84]. By means of fluorescent immunostaining of 5-methylcytidine, the authors determined that, when compared to control unperturbed embryos, arsenic-treated embryos displayed abnormal hypomethylation in the trunk and tail at early developmental stages. This trend was overturned during the remaining phases of development and aberrant hypermethylation was detected across the whole embryo body, especially in the tail [84]. Notably, this information highlights the versatility of the zebrafish model for inspecting changes in the overall DNA methylation pattern among distinct spatial sectors of a whole organism.

Paradoxically, however, the global DNA methylation level could not be an informative epigenetic marker, being the epigenetic effects driven by site-specific changes that may be obscured on a global scale, as highlighted by several studies. Among these, Bouwmeester et al. [85] performed a systematic screening and exposing of fish embryos to subtoxic concentrations of a range of environmentally relevant xenobiotics of known epigenetic effects, which potentially play a role in developmental origins of adult diseases. The authors found that the bulk genomic methylation level did not vary in embryos exposed to any of the test compounds. Nevertheless, pyrosequencing analysis of methylation in the promoter of selected informative target genes displayed significant differences between control and exposed embryos [85]. For instance, the estrogenic compounds diethylstilbestrol and 17α-ethynylestradiol induced reproducible hypomethylation in the CpG island of the germline-specific marker *vasa*, while the metals Ni and Cd both induced hypermethylation in the same genomic region. It is worth mentioning that a subset of the tested compounds, which includes bisphenol-A and perfluorooctanoic acid, specifically affected site-specific DNA methylation at concentrations unable to inflict overt adverse phenotypes. Altogether, these findings not only reaffirm the applicability of the zebrafish embryo as a valuable screening model for epigenetic modifications after xenobiotic exposure, but also suggest that in these assays opposed locus-specific methylation changes could balance each other, not being reproduced on the global genome-scale methylation level.

Identification of changes in gene-specific methylation represents a fundamental issue in the emerging field of enantioselective environmental epigenetics. In this connection, several pollutants contain a chiral structure consisting of enantiomers that, despite having identical physical–chemical properties, selectively impinge on biological mechanisms [86–89]. To date, only a single report has shed new light on the toxicity of chiral compounds from the perspective of enantioselective epigenetic regulation in a developing organism, and zebrafish was the model successfully used [90]. In this study, the authors focused on the modification of DNA methylation induced by the R-(−) and S-(+) enantiomers of fipronil, a *n*-phenylpyrazole insecticide [91]. They found that the S-(+) fipronil exerted significantly greater developmental toxicity compared to the R-(−) enantiomer, resulting in a massive increase in both global and gene-specific DNA methylation [90]. In this analysis, no fewer than 22 molecular pathways each containing more than five hypermethylated genes were identified by the KEGG database, and seven of these pathways were strictly associated with pivotal developmental processes [90]. As expected, five out of seven randomly selected genes containing hypermethylated promoters were confirmed to be transcriptionally downregulated to a greater extent by S-(+) fipronil, rather than R-(−) fipronil, exposure.

## Environmental effects mediated by Dnmts

A few recent studies have suggested that pollutant exposure could induce alteration in DNA methylation patterns by disturbing *dnmt* gene expression during zebrafish embryogenesis. Among these, a couple of reports described the impact of 2,3,7,8-tetrachlorodibenzo-*p*-dioxin (TCDD) on DNA methylation of embryos, larvae, and adult zebrafish [92, 93]. TCDD is a halogenated polycyclic hydrocarbon acting as a ligand for the aryl hydrocarbon receptor (AHR) transcription factor, which plays a role in mediating the toxic developmental effects of TCDD in zebrafish. Indeed, binding of TCDD allows nuclear translocation and recruitment of AHR to xenobiotic response elements in the promoter regions of a variety of target genes [94–97].

In accordance with findings from other groups [85], both the studies mentioned concordantly highlighted that, although TCDD did not affect the overall amount of 5-methylcytosine during development, specific methylation of the CpG islands in the promoter of AHR target genes was either unchanged or differentially affected. For instance, hypomethylation was observed in 11 out of 22 CG dinucleotides within the *cfos* promoter, while 14 out of 34 CG sites were hypermethylated in the *ahrra* promoter [93].

Interestingly, these alterations have been supposed to be dependent upon TCDD-induced deregulation of *dnmt* gene expression. Indeed, TCDD exposure during early embryogenesis provoked developmental stage-specific upregulation of *dnmt1* and *dnmt3b2*, coupled to downregulation of *dnmt3a1*, *dnmt3b1*, and *dnmt3b4* [93]. The specificity of these effects is further supported by the observation that expression of *dnmt3a2* and *dnmt3b3* was not affected by TCDD treatment [93]. These findings strongly suggest that TCDD could impact both establishment and maintenance of DNA methylation patterns of genomic loci not necessarily restricted to AHR targets.

Another relevant study, focusing on the epigenetic effect evoked by lead (Pb), also advocated a direct relationship between changes in Dnmt activity/expression and the DNA methylation level of zebrafish embryos [98]. The authors first determined that Pb exposure modulates the activity of the maintenance Dnmt enzyme via non-competitive inhibition, in vitro. They also described the alteration of expression patterns of the de novo Dnmt enzymes during development of zebrafish Pb-exposed embryos, which in turn displayed overall DNA hypomethylation [98].

By contrast, divergent findings come from other studies indicating that gene expression and activity of the various Dnmts are not affected by exposure to environmental pollutants. For example, treatment of developing zebrafish embryos with benzo[*a*]pyrene, a potent DNA-hypomethylating compound, did not alter either transcriptional or enzymatic activity of Dnmts [62]. However, it should be emphasized that the authors measured the global activities from all the Dnmt isozymes in nuclear extracts derived from benzo[*a*]pyrene-exposed embryos, so that potential compensative changes among the activity of individual Dnmts cannot be excluded. Indeed, a more recent study confirmed that the mRNA abundance of the various *dnmts* was differentially altered in benzo[*a*]pyrene-treated zebrafish embryos at 24 h post-fertilization [99]. In particular, while the transcript levels of *dnmt3b2* were elevated, those of *dnmt1* and *dnmt3a2* were significantly reduced, and those of *dnmta1* and *dnmtb1* were not affected [99].

Beyond this, Fang et al. [62] also noted that benzo[*a*]pyrene exposure substantially stimulated activity, but not gene transcription, of the glycine *N*-methyltransferase (Gnmt) enzyme. Gnmt is probably the most important enzyme regulating the metabolic transmethylation flux in animal organisms, where it catalyzes the transfer of a methyl group from *S*-adenosyl-methionine (SAM) to glycine-forming *S*-adenosyl-homocysteine [100]. Interestingly, there is a functional relationship between Gnmt expression and DNA methylation, mediated by SAM concentrations [101, 102]. Based on this, an increase in Gnmt activity, in the absence of changes in Dnmt activity, could account for the decreased SAM amount, which in turn could explain the loss of global DNA methylation in benzo[*a*]pyrene-exposed embryos.

Zebrafish, like other aquatic organisms, are likely exposed to multiple environmental stressors, which could impose additive effects on the epigenomic landscape. Following this consideration, Dorts et al. [103] reported that the combination of heat stress and copper exposure provokes synergistic adverse developmental effects upregulating the expression of all the *dnmt3* genes without apparent changes in the global DNA methylation level. Once again, this finding does not necessarily mean that DNA methylation modifications did not occur. Therefore, although the authors did not determine site-specific DNA methylation, a potential effect on the establishment of DNA methylation patterns in the promoter of selected genes cannot be excluded.

## Transgenerational inheritance of DNA methylation by environmental compounds

Although environmental stressors acting on somatic cells can potentially influence the epigenetic program of the individual developing organism exposed, epigenetic alterations can be propagated to subsequent generations through the germline, even in the absence of further stressor exposures [104]. With so far very few though intriguing studies, zebrafish is also emerging as a useful model for studying long-term transgenerational effects of environmental factors on both epigenetic and phenotypic variations.

In one of these studies, adult zebrafish females were fed with a diet enriched in either TCDD, methylmercury (MeHg), or 5-aza-2′-deoxycytidine, and offspring from two subsequent generations was assessed for changes in DNA methylation [92]. Surprisingly, the authors observed weak evidence of alteration in the methylome of the F2 individuals, concluding that the compounds mentioned did not cause transgenerational effects in zebrafish. However, at least two technical flaws in the experimental strategy employed could have accidentally distorted the interpretation of their results. First, it should be noted that the exposure window did not include epigenetic reprogramming of DNA methylation occurring during early embryogenesis, which is critical for transgenerational effects. In addition, only female individuals were exposed to the above-mentioned compounds, probably based on the evidence of a previous study by other authors hypothesizing the exclusion of potential effects on the male germline [52]. In particular, these authors explored the DNA methylation dynamics of fifteen selected genes in maternal haploid parthenogenic embryos, which do not have paternal genome contribution. Strikingly, they found that the mentioned genes underwent reprogramming timely to an extent that was indistinguishable from control embryos derived from normal mating [52]. This finding suggests that the maternal genome/transcriptome/proteome in the embryo is sufficient for instructing DNA methylation reprogramming. Although intriguing, this conclusion suffers from two main weakness, viz. the extremely exiguous set of genes explored and the absence of data about the DNA methylation dynamics of noncoding loci. Following this line of reasoning, most paternal transgenerational effects could be potentially conveyed through the noncoding genome fraction.

Some support to this theory has been lent by observations from a very recent study highlighting the influence of developmental exposure to MeHg on the inheritance of phenotypic malformations in correlation with epimutations consisting in reproducible patterns of differential DNA methylation [105]. In particular, the authors noted that fertilized eggs of the F0 generation exposed to MeHg until 24 h post-fertilization show hyperactivity, visual deficits, and altered retinal electrophysiology [105]. Strikingly, although these fish, as well as their offspring of the F1 and F2 generations, were reared without additional exposures to MeHg for their entire life cycle, the F2 individuals displayed exactly the same phenotypic defects mentioned above. Compared to unexposed controls, the sperm DNA isolated from the F2 fish ancestrally exposed to MeHg did contain a highly reproducible set of differentially methylated regions. Intriguingly, although a number of these regions map within the promoter of genes that may correlate with the behavioral phenotypes observed, the vast majority of differentially methylated sites did not have gene associations [105]. Such a captivating finding could suggest that these regions of noncoding genome are probably involved in the regulation of gene expression by either *cis*-regulatory mechanisms or production of noncoding RNA.

In a coeval study performed by a distinct group, the authors assessed the transgenerational effects of two distinct compounds, the well-known Dnmt1 inhibitor 5-azacytidine [106, 107] and the plasticizer derivative mono(2-ethylhexyl)phthalate (MEHP), which is ubiquitously present in the environment [108]. It is worth mentioning that in this study only fertilized eggs of the F0 generation were exposed once in a lifetime, until 6 days post-fertilization, to 5-azacytidine or MEHP at concentrations unable to elicit detectable adverse effects on development [109]. Despite this, both compounds altered *dnmt* gene expression and DNA methylation level to a different extent in the directly exposed individuals. Comparative genome-wide analysis of the DNA methylation patterns of the offspring of these fish and unperturbed controls at the F0, F1, and F2 generations indicated that methylation changes provoked by ancestral exposure to the compounds mentioned are persistent across generations. Even in this case, in perfect agreement with the finding described above, differential methylation was frequently found outside gene bodies and promoters, being enriched at distal noncoding regions that could have relevant regulatory roles. This interesting hypothesis is further supported by the evolutionary conservation of these genomic regions across vertebrate organisms, including humans [110].

## Modification of additional epigenetic profiles by environmental compounds

As summarized throughout this review, the vast majority of the environmental epigenetic studies in zebrafish interrogated DNA methylation. However, as outlined by studies using other organisms, additional epigenetic factors are equally important for sensing of environmental stressors. To date, limited studies in zebrafish have highlighted the variation in epigenetic marks, such as miRNAs and histone post-translational modifications, following exposure to toxicants or pollutants. For example, a recent study indicated that the teratogenic effects of sublethal concentrations of ethanol on zebrafish embryogenesis are mediated by a major increase in the abundance of a specific subset of miRNAs, which the authors proposed to be a signature for ethanol-induced toxicity in vertebrates [111].

In a similar study, microarray analysis was applied to assess the differential variation of a panel of miRNAs following exposure of zebrafish embryos to perfluorooctane sulfonate, a widely distributed environmentally organic compound, which has been found to cause developmental toxicity [112, 113]. Being the predicted targets of these miRNAs involved in a broad spectrum of developmental, cellular, and metabolic processes, this preliminary study could address the epigenetic explanation of toxicity induced by the compound mentioned.

An additional noteworthy study evaluated the genome-wide occupancy of H3K27 and H3K9 histone trimethylation following exposure of developing zebrafish embryos to 3-deazaneplanocin-A (DZNep), an anti-cancer drug that unselectively inhibits EZH2 histone methyltransferase of the polycomb repressive complex 2 responsible for H3K27 methylation [114, 115]. Interestingly, DZNep exposure provoked a dose-dependent depletion and alteration in distribution of H3K27me3 and H3K9me3 from a substantial number of gene promoters. These epigenetic variations were associated with severe neuronal and cranial malformations in the exposed fish, although they unexpectedly did not result in significant changes in gene expression levels. An explanation for this paradoxical observation could be that, as noted by the authors, DZNep does not prevent de novo acquisition of histone lysine methylation [114].

## Toward a deeper understanding of mode(s) of epigenetic inheritance

Presently, experimental clues suggesting that epigenetic marks acquired by the germline are perpetuated to fish of subsequent generations remain quite limited. As described in the previous sections, DNA methylation actually represents the best-characterized epigenetic factor to be involved in transmission of epigenetic information. The paradigm of epigenetic inheritance is certainly the genomic imprinting that mediates paternal or maternal allelic transmission of specific DNA methylation patterns [116]. An auxiliary example has been provided by studies on the tonguefish *Cynoglossus semilaevis*. This teleost fish employs a primary mechanism of sex determination based on chromosome inheritance, whereas female and male individuals bear either a ZW or ZZ chromosome configuration, respectively [117, 118]. The complex mechanism responsible for male sex determination relies on a gene regulatory network triggered by the Z-linked *dmrt1* gene, which is repressed and heavily methylated in the promoter region during gonadal differentiation of female individuals [119].

Interestingly, a fraction of ZW females is spontaneously sex-reversed into phenotypic males, referred to as pseudomales, which can mate with normal females to produce viable offspring [119]. More importantly, the extent of sex reversal responds to changes in environmental temperature, and it is inherited by the subsequent generation reared in normal conditions [119]. Consistently, the sex-reversed pseudomales (as well as normal males) show high gonadal *dmrt1* expression coupled to extremely low methylation levels of the *dmrt1* promoter [120]. Although the cause–effect relationship between differential DNA methylation and sex reversal remains to be clarified, this study clearly highlights that DNA methylation plays a fundamental role in transgenerational epigenetic inheritance in tonguefish.

Similar DNA methylation-based mechanisms probably regulate transgenerational epigenetic inheritance also in zebrafish, which has been postulated to have female dominant (ZW/ZZ) sex determination system [121]. Moreover, the genomic distribution of CpG islands and the percentage of 5-methylcytosine are both generally conserved between tonguefish and zebrafish [120].

An interesting line of questioning to pursue in the future would be to correlate the inheritance of environmentally altered DNA methylation patterns with changes in the expression of the gene toolkit responsible for DNA methylation and demethylation. So far, very scarce and confusing information is available on this point. For example, Olsvik et al. reported that the F2 offspring of F0 adult female zebrafish exposed to MeHg has only modest effects on both DNA methylation and *dnmts* expression, even though a number of site-specific methylation changes were detected in the F1 fish [92]. These data are difficult to interpret because the experimental design conceived by the authors (breeding of MeHg-treated F0 female with non-exposed F0 male fish) precluded examination of the paternal chromatin role in the transmission of DNA methylation patterns from one generation to the next. A pertinent study in this trajectory reported the transgenerational inheritance of heart disorders in the F2 offspring derived from F0 male adult fish exposed to bisphenol-A [122]. The aberrant phenotypes were consistently associated with downregulation of several genes involved in cardiac embryo development [122]. Unfortunately, although this finding suggests that the epigenetic landscape of these genes have probably changed, the authors did not address DNA methylation at their promoters. Indirect complementary observations come from a distinct study highlighting that chronic exposure to bisphenol-A, at concentrations that do not produce any obvious malformations, alters the expression of *dnmt1*, *dnmt3b3*, *dnmt3b4* genes across two generations of fish [123]. Future systematic analysis should uncover the specific contribution for each of these genes to transgenerational epigenetic inheritance.

Beyond the DNA methylation machinery, a series of compelling evidence also suggested that retention of prepatterned histone modifications in sperm chromatin could have instructive roles for the developmental program. Unlike the mammalian male gametes, the mature zebrafish sperm chromatin lacks protamine, transition proteins, and testis-specific histone variants [124]. Nonetheless, chromatin compaction is entrusted to hypoacetylated nucleosomal histones and higher amounts of linker histone compared to somatic cells [124]. Notably, coincidence of several permissive and repressive histone modifications has been found in blocks of multivalent sperm chromatin containing developmental genes with regulatory functions, constituting a mark predictive for their embryonic expression [124, 125]. Relevant to this idea, the histone modifications mentioned are not erased at fertilization, persisting in the early developing embryo [125]. Altogether, these findings strongly support a model of transgenerational epigenetic inheritance along the paternal lineage in zebrafish.

On the other hand, this model apparently clashes with earlier antithetic observations, indicating that histone modification patterns are initially not associated with the chromatin of the early developing zebrafish embryo, emerging following zygotic genome activation [126]. Such a negative result could be explained by the insufficient sensitivity of the detection assay used by the authors. Indeed, early embryonic stages are technically challenging to examine due to the low level of modified histones. In addition, it could be speculated that the overall amount of histone modifications is partially erased or diluted or replaced by other epigenetic marks in the embryo before the onset of zygotic genome activation.

More recently, a number of attractive studies in mice suggested regulatory roles for further epigenetic factors, such as noncoding RNA and three-dimensional chromatin architecture, in epigenetic transgenerational inheritance [127–129]. Although similar studies have not yet been accomplished in zebrafish, it could be syllogistically inferred that the multidimensional coordination of distinct epigenetic processes likely governs the environmentally induced epigenetic transgenerational inheritance phenomenon.

## Conclusions

In this review, we have reported and discussed recent evidence that strongly supports the idea that the zebrafish can be a valuable animal model for exploring both individual and transgenerational epigenetic variations induced by a wide variety of environmental stimuli. So far, experimental investigation has focused mostly on DNA methylation due to the functional link between epigenetic (re)programming and DNA methylation. Future studies are required to adequately elucidate the roles played by additional epigenetic processes involving histone modifications, noncoding RNA, and chromatin structure. Clearly, more research on this field using zebrafish is warranted, in order to fully understand the impact of the environment on the epigenome, and in turn the phenotype, of vertebrate organisms.

## Abbreviations

AHR: aryl hydrocarbon receptor
Dnmt: DNA methyltransferase
DZNep: 3-deazaneplanocin-A
Gnmt: glycine *N*-methyltransferase
H3K9me3: histone H3 lysine 9 trimethylation
H3K27me3: histone H3 lysine 27 trimethylation
KEGG: Kyoto Encyclopedia of Genes and Genomes
MeHg: methylmercury
MEHP: mono(2-ethylhexyl)phthalate
miRNA: microRNA
SAM: *S*-adenosyl-methionine
TCDD: 2,3,7,8-tetrachlorodibenzo-*p*-dioxin
Tet: ten-eleven translocation protein

## References

[1] Jirtle R, Bird A (2003). Epigenetic regulation of gene expression: how the genome integrates intrinsic and environmental signals. Nat Genet.

[2] Szyf M (2011). The early life social environment and DNA methylation: DNA methylation mediating the long-term impact of social environments early in life. Epigenetics.

[3] Cortessis VK, Thomas DC, Levine AJ, Breton CV, Mack TM, Siegmund KD, Haile RW, Laird PW (2012). Environmental epigenetics: prospects for studying epigenetic mediation of exposure–response relationships. Hum Genet.

[4] Bird A (2002). DNA methylation patterns and epigenetic memory. Genes Dev.

[5] Kouzarides T (2007). Chromatin modifications and their function. Cell.

[6] Cui P, Zhang L, Lin Q, Ding F, Xin C, Fang X, Hu S, Yu J (2010). A novel mechanism of epigenetic regulation: nucleosome-space occupancy. Biochem Biophys Res Commun.

[7] Mercer TR, Mattick JS (2013). Structure and function of long noncoding RNAs in epigenetic regulation. Nat Struct Mol Biol.

[8] Talbert PB, Henikoff S (2014). Environmental responses mediated by histone variants. Trends Cell Biol.

[9] Garfield DA, Runcie DE, Babbitt CC, Haygood R, Nielsen WJ, Wray GA (2013). The impact of gene expression variation on the robustness and evolvability of a developmental gene regulatory network. PLoS Biol.

[10] Cavalieri V, Spinelli G (2014). Early asymmetric cues triggering the dorsal/ventral gene regulatory network of the sea urchin embryo. Elife.

[11] Boettiger AN, Bintu B, Moffitt JR, Wang S, Beliveau BJ, Fudenberg G, Imakaev M, Mirny LA, Wu CT, Zhuang X (2016). Super-resolution imaging reveals distinct chromatin folding for different epigenetic states. Nature.

[12] Turner BM (2009). Epigenetic responses to environmental change and their evolutionary implications. Philos Trans R Soc Lond B Biol Sci.

[13] Skinner MK, Gurerrero-Bosagna C, Haque MM, Nilsson EE, Koop JA, Knutie SA, Clayton DH (2014). Epigenetics and the evolution of Darwin’s Finches. Genome Biol Evol..

[14] Youngson NA, Whitelaw E (2008). Transgenerational epigenetic effects. Annu Rev Genomics Hum Genet.

[15] Gapp K, von Ziegler L, Tweedie-Cullen RY, Mansuy IM (2014). Early life epigenetic programming and transmission of stress-induced traits in mammals: how and when can environmental factors influence traits and their transgenerational inheritance?. BioEssays.

[16] Bertoldo MJ, Locatelli Y, O’Neill C, Mermillod P (2015). Impacts of and interactions between environmental stress and epigenetic programming during early embryo development. Reprod Fertil Dev.

[17] Ci W, Liu J (2015). Programming and inheritance of parental DNA methylomes in vertebrates. Physiology (Bethesda).

[18] Di Caro V, Cavalieri V, Melfi R, Spinelli G (2007). Constitutive promoter occupancy by the MBF-1 activator and chromatin modification of the developmental regulated sea urchin alpha-H2A histone gene. J Mol Biol.

[19] Feeney A, Nilsson E, Skinner MK (2014). Epigenetics and transgenerational inheritance in domesticated farm animals. J Anim Sci Biotechnol.

[20] Cavalieri V, Spinelli G (2015). Ectopic hbox12 expression evoked by histone deacetylase inhibition disrupts axial specification of the sea urchin embryo. PLoS ONE.

[21] Bonasio R (2015). The expanding epigenetic landscape of non-model organisms. J Exp Biol.

[22] Leroux S, Gourichon D, Leterrier C, Labrune Y, Coustham V, Rivière S, Zerjal T, Coville JL, Morisson M, Minvielle F, Pitel F (2017). Embryonic environment and transgenerational effects in quail. Genet Sel Evol.

[23] Razin A, Riggs AD (1980). DNA methylation and gene function. Science.

[24] Lister R, Pelizzola M, Dowen RH, Hawkins RD, Hon G, Tonti-Filippini J, Nery JR, Lee L, Ye Z, Ngo QM, Edsall L, Antosiewicz-Bourget J, Stewart R, Ruotti V, Millar AH, Thomson JA, Ren B, Ecker JR (2009). Human DNA methylomes at base resolution show widespread epigenomic differences. Nature.

[25] Jones PA (2012). Functions of DNA methylation: islands, start sites, gene bodies and beyond. Nat Rev Genet.

[26] Riggs AD (2002). X chromosome inactivation, differentiation, and DNA methylation revisited, with a tribute to Susumu Ohno. Cytogenet Genome Res.

[27] Lindroth AM, Cao X, Jackson JP, Zilberman D, McCallum CM, Henikoff S, Jacobsen SE (2001). Requirement of CHROMOMETHYLASE3 for maintenance of CpXpG methylation. Science.

[28] Lister R, Pelizzola M, Kida YS, Hawkins RD, Nery JR, Hon G, Antosiewicz-Bourget J, O’Malley R, Castanon R, Klugman S, Downes M, Yu R, Stewart R, Ren B, Thomson JA, Evans RM, Ecker JR (2011). Hotspots of aberrant epigenomic reprogramming in human induced pluripotent stem cells. Nature.

[29] Varley KE, Gertz J, Bowling KM, Parker SL, Reddy TE, Pauli-Behn F, Cross MK, Williams BA, Stamatoyannopoulos JA, Crawford GE, Absher DM, Wold BJ, Myers RM (2013). Dynamic DNA methylation across diverse human cell lines and tissues. Genome Res.

[30] Goll MG, Bestor TH (2005). Eukaryotic cytosine methyltransferases. Annu Rev Biochem.

[31] Edwards JR, Yarychkivska O, Boulard M, Bestor TH (2017). DNA methylation and DNA methyltransferases. Epigenet Chromatin.

[32] Kagiwada S, Kurimoto K, Hirota T, Yamaji M, Saitou M (2013). Replication-coupled passive DNA demethylation for the erasure of genome imprints in mice. EMBO J.

[33] Arand J, Wossidlo M, Lepikhov K, Peat JR, Reik W, Walter J (2015). Selective impairment of methylation maintenance is the major cause of DNA methylation reprogramming in the early embryo. Epigenet Chromatin.

[34] Dean W (2016). Pathways of DNA demethylation. Adv Exp Med Biol.

[35] Tahiliani M, Peng Koh K, Shen Y, Pastor WA, Bandukwala H, Brudno Y, Agarwal S, Iyer LM, Liu DR, Aravind L, Rao A (2009). Conversion of 5-methylcytosineto 5-hydroxymethylcytosine in mammalian DNA by MLL partner TET1. Science.

[36] Bhutani N, Burns DM, Blau HM (2011). DNA demethylation dynamics. Cell.

[37] Santos F, Peat J, Burgess H, Rada C, Reik W, Dean W (2013). Active demethylation in mouse zygotes involves cytosine deamination and base excision repair. Epigenet Chromatin.

[38] Feil R (2009). Epigenetic asymmetry in the zygote and mammalian development. Int J Dev Biol.

[39] Geiman TM, Muegge K (2010). DNA methylation in early development. Mol Reprod Dev.

[40] Reis Silva AR, Adenot P, Daniel N, Archilla C, Peynot N, Lucci CM, Beaujean N, Duranthon V (2011). Dynamics of DNA methylation levels in maternal and paternal rabbit genomes after fertilization. Epigenetics.

[41] Bogdanović O, Gómez-Skarmeta JL (2014). Embryonic DNA methylation: insights from the genomics era. Brief Funct Genomics.

[42] Heras S, Smits K, De Schauwer C, Van Soom A (2017). Dynamics of 5-methylcytosine and 5-hydroxymethylcytosine during pronuclear development in equine zygotes produced by ICSI. Epigenet Chromatin.

[43] Engeszer RE, Patterson LB, Rao AA, Parichy DM (2007). Zebrafish in the wild: a review of natural history and new notes from the field. Zebrafish.

[44] Gonzales JM (2012). Preliminary evaluation on the effects of feeds on the growth and early reproductive performance of zebrafish (Danio rerio). J Am Assoc Lab Anim Sci.

[45] Kimmel CB, Ballard WW, Kimmel SR, Ullmann B, Schilling TF (1995). Stages of embryonic development of the zebrafish. Dev Dyn.

[46] Melani C, Campana M, Lombardot B, Rizzi B, Veronesi F, Zanella C, Bourgine P, Mikula K, Peyriéras N, Sarti A (2007). Cells tracking in a live zebrafish embryo. Conf Proc IEEE Eng Med Biol Soc.

[47] Godinho L (2011). Live imaging of zebrafish development. Cold Spring Harb Protoc.

[48] Ali S, Aalders J, Richardson MK (2014). Teratological effects of a panel of sixty water-soluble toxicants on zebrafish development. Zebrafish.

[49] Howe K, Clark MD, Torroja CF, Torrance J, Berthelot C, Muffato M, Collins JE, Humphray S, McLaren K, Matthews L, McLaren S, Sealy I, Caccamo M, Churcher C, Scott C, Barrett JC, Koch R, Rauch GJ, White S, Chow W, Kilian B, Quintais LT, Guerra-Assunção JA, Zhou Y, Gu Y, Yen J, Vogel JH, Eyre T, Redmond S, Banerjee R, Chi J, Fu B, Langley E, Maguire SF, Laird GK, Lloyd D, Kenyon E, Donaldson S, Sehra H, Almeida-King J, Loveland J, Trevanion S, Jones M, Quail M, Willey D, Hunt A, Burton J, Sims S, McLay K, Plumb B, Davis J, Clee C, Oliver K, Clark R, Riddle C, Elliot D, Threadgold G, Harden G, Ware D, Begum S, Mortimore B, Kerry G, Heath P, Phillimore B, Tracey A, Corby N, Dunn M, Johnson C, Wood J, Clark S, Pelan S, Griffiths G, Smith M, Glithero R, Howden P, Barker N, Lloyd C, Stevens C, Harley J, Holt K, Panagiotidis G, Lovell J, Beasley H, Henderson C, Gordon D, Auger K, Wright D, Collins J, Raisen C, Dyer L, Leung K, Robertson L, Ambridge K, Leongamornlert D, McGuire S, Gilderthorp R, Griffiths C, Manthravadi D, Nichol S, Barker G, Whitehead S, Kay M, Brown J, Murnane C, Gray E, Humphries M, Sycamore N, Barker D, Saunders D, Wallis J, Babbage A, Hammond S, Mashreghi-Mohammadi M, Barr L, Martin S, Wray P, Ellington A, Matthews N, Ellwood M, Woodmansey R, Clark G, Cooper J, Tromans A, Grafham D, Skuce C, Pandian R, Andrews R, Harrison E, Kimberley A, Garnett J, Fosker N, Hall R, Garner P, Kelly D, Bird C, Palmer S, Gehring I, Berger A, Dooley CM, Ersan-Ürün Z, Eser C, Geiger H, Geisler M, Karotki L, Kirn A, Konantz J, Konantz M, Oberländer M, Rudolph-Geiger S, Teucke M, Lanz C, Raddatz G, Osoegawa K, Zhu B, Rapp A, Widaa S, Langford C, Yang F, Schuster SC, Carter NP, Harrow J, Ning Z, Herrero J, Searle SM, Enright A, Geisler R, Plasterk RH, Lee C, Westerfield M, de Jong PJ, Zon LI, Postlethwait JH, Nüsslein-Volhard C, Hubbard TJ, Roest Crollius H, Rogers J, Stemple DL (2013). The zebrafish reference genome sequence and its relationship to the human genome. Nature.

[50] Amsterdam A, Nissen RM, Sun Z, Swindell EC, Farrington S, Hopkins N (2004). Identification of 315 genes essential for early zebrafish development. Proc Natl Acad Sci USA.

[51] Jiang L, Zhang J, Wang JJ, Wang L, Zhang L, Li G, Yang X, Ma X, Sun X, Cai J, Zhang J, Huang X, Yu M, Wang X, Liu F, Wu CI, He C, Zhang B, Ci W, Liu J (2013). Sperm, but not oocyte, DNA methylome is inherited by zebrafish early embryos. Cell.

[52] Potok ME, Nix DA, Parnell TJ, Cairns BR (2013). Reprogramming the maternal zebrafish genome after fertilization to match the paternal methylation pattern. Cell.

[53] Varriale A, Bernardi G (2006). DNA methylation and body temperature in fishes. Gene.

[54] Feng S, Cokus SJ, Zhang X, Chen PY, Bostick M, Goll MG, Hetzel J, Jain J, Strauss SH, Halpern ME, Ukomadu C, Sadler KC, Pradhan S, Pellegrini M, Jacobsen SE (2010). Conservation and divergence of methylation patterning in plants and animals. Proc Natl Acad Sci USA.

[55] Zemach A, McDaniel IE, Silva P, Zilberman D (2010). Genome-wide evolutionary analysis of eukaryotic DNA methylation. Science.

[56] Goll MG, Halpern ME (2011). DNA methylation in zebrafish. Prog Mol Biol Transl Sci.

[57] Streisinger G, Walker C, Dower N, Knauber D, Singer F (1981). Production of clones of homozygous diploid zebra fish (*Brachydanio rerio*). Nature.

[58] Head JA (2014). Patterns of DNA methylation in animals: an ecotoxicological perspective. Integr Comp Biol.

[59] Mhanni AA, McGowan RA (2004). Global changes in genomic methylation levels during early development of the zebrafish embryo. Dev Genes Evol.

[60] MacKay AB, Mhanni AA, McGowan RA, Krone PH (2007). Immunological detection of changes in genomic DNA methylation during early zebrafish development. Genome.

[61] Wu SF, Zhang H, Hammoud SS, Potok M, Nix DA, Jones DA, Cairns BR (2011). DNA methylation profiling in zebrafish. Methods Cell Biol.

[62] Fang X, Corrales J, Thornton C, Scheffler BE, Willett KL (2013). Global and gene specific DNA methylation changes during zebrafish development. Comp Biochem Physiol B: Biochem Mol Biol.

[63] Jabbari K, Cacciò S, Pais de Barros JP, Desgrès J, Bernardi G (1997). Evolutionary changes in CpG and methylation levels in the genome of vertebrates. Gene.

[64] Mhanni AA, Yoder JA, Dubesky C, McGowan RA (2001). Cloning and sequence analysis of a zebrafish cDNA encoding DNA (cytosine-5)-methyltransferase-1. Genesis.

[65] Shimoda N, Yamakoshi K, Miyake A, Takeda H (2005). Identification of a gene required for de novo DNA methylation of the zebrafish no tail gene. Dev Dyn.

[66] Campos C, Valente LMP, Fernandes JMO (2012). Molecular evolution of zebrafish dnmt3 genes and thermal plasticity of their expression during embryonic development. Gene.

[67] Ge L, Zhang RP, Wan F, Guo DY, Wang P, Xiang LX, Shao JZ (2014). TET2 plays an essential role in erythropoiesis by regulating lineage-specific genes via DNA oxidative demethylation in a zebrafish model. Mol Cell Biol.

[68] Bogdanović O, Smits AH, de la Calle ME, Tena JJ, Ford E, Williams R, Senanayake U, Schultz MD, Hontelez S, van Kruijsbergen I, Rayon T, Gnerlich F, Carell T, Veenstra GJ, Manzanares M, Sauka-Spengler T, Ecker JR, Vermeulen M, Gómez-Skarmeta JL, Lister R (2016). Active DNA demethylation at enhancers during the vertebrate phylotypic period. Nat Genet.

[69] Wilson VL, Jones PA (1983). Inhibition of DNA methylation by chemical carcinogens in vitro. Cell.

[70] Wojciechowski MF, Meehan T (1984). Inhibition of DNA methyltransferases in vitro by benzo[*a*]pyrene diol epoxide-modified substrates. J Biol Chem.

[71] Sadikovic B, Rodenhiser DI (2006). Benzopyrene exposure disrupts DNA methylation and growth dynamics in breast cancer cells. Toxicol Appl Pharmacol.

[72] Yoon C, Kawakami K, Hopkins N (1997). Zebrafish vasa homologue RNA is localized to the cleavage planes of 2- and 4-cell-stage embryos and is expressed in the primordial germ cells. Development.

[73] Knaut H, Pelegri F, Bohmann K, Schwarz H, Nusslein-Volhard C (2000). Zebrafish vasa RNA but not its protein is a component of the germ plasm and segregates asymmetrically before germline specification. J Cell Biol.

[74] Li M, Hong N, Xu H, Yi M, Li C, Gui J, Hong Y (2009). Medaka vasa is required for migration but not survival of primordial germ cells. Mech Dev.

[75] Abbott DH, Barnett DK, Bruns CM, Dumesic DA (2005). Androgen excess fetal programming of female reproduction: a developmental aetiology for polycystic ovary syndrome?. Hum Reprod Update.

[76] Ramezani Tehrani F, Noroozzadeh M, Zahediasl S, Ghasemi A, Piryaei A, Azizi F (2013). Prenatal testosterone exposure worsen the reproductive performance of male rat at adulthood. PLoS ONE.

[77] Xu N, Chua AK, Jiang H, Liu NA, Goodarzi MO (2014). Early embryonic androgen exposure induces transgenerational epigenetic and metabolic changes. Mol Endocrinol.

[78] Padmanabhan V, Manikkam M, Recabarren S, Foster D (2006). Prenatal testosterone excess programs reproductive and metabolic dysfunction in the female. Mol Cell Endocrinol.

[79] Liu Y, Yuan C, Chen S, Zheng Y, Zhang Y, Gao J, Wang Z (2014). Global and cyp19a1a gene specific DNA methylation in gonads of adult rare minnow *Gobiocypris rarus* under bisphenol A exposure. Aquat Toxicol.

[80] Nemec MD, Holson JF, Farr CH, Hood RD (1998). Developmental toxicity assessment of arsenic acid in mice and rabbits. Reprod Toxicol.

[81] Rodriguez VM, Carrizales L, Mendoza MS, Fajardo OR, Giordano M (2002). Effects of sodium arsenite exposure on development and behavior in the rat. Neurotoxicol Teratol.

[82] Tchounwou PB, Centeno JA, Patlolla AK (2004). Arsenic toxicity, mutagenesis, and carcinogenesis—a health risk assessment and management approach. Mol Cell Biochem.

[83] Hill DS, Wlodarczyk BJ, Finnell RH (2008). Reproductive consequences of oral arsenate exposure during pregnancy in a mouse model. Birth Defects Res B Dev Reprod Toxicol.

[84] Li D, Lu C, Wang J, Hu W, Cao Z, Sun D, Xia H, Ma X (2009). Developmental mechanisms of arsenite toxicity in zebrafish (*Danio rerio*) embryos. Aquat Toxicol.

[85] Bouwmeester MC, Ruiter S, Lommelaars T, Sippel J, Hodemaekers HM, van den Brandhof EJ, Pennings JL, Kamstra JH, Jelinek J, Issa JP, Legler J, van der Ven LT (2016). Zebrafish embryos as a screen for DNA methylation modifications after compound exposure. Toxicol Appl Pharmacol.

[86] Lewis DL, Garrison AW, Wommack KE, Whittemore A, Steudler P, Melillo J (1999). Influence of environmental changes on degradation of chiral pollutants in soils. Nature.

[87] Chen F, Zhang Q, Wang C, Lu Y, Zhao M (2012). Enantioselectivity in estrogenicity of the organochlorine insecticide acetofenate in human trophoblast and MCF-7 cells. Reprod Toxicol.

[88] Zhao M, Zhang Y, Zhuang S, Zhang Q, Lu C, Liu W (2014). Disruption of the hormonal network and the enantioselectivity of bifenthrin in trophoblast: maternal–fetal health risk of chiral pesticides. Environ Sci Technol.

[89] Zhuang S, Zhang Z, Zhang W, Bao L, Xu C, Zhang H (2015). Enantioselective developmental toxicity and immunotoxicity of pyraclofos toward zebrafish (*Danio rerio*). Aquat Toxicol.

[90] Qian Y, Wang C, Wang J, Zhang X, Zhou Z, Zhao M, Lu C (2017). Fipronil-induced enantioselective developmental toxicity to zebrafish embryo-larvae involves changes in DNA methylation. Sci Rep.

[91] Teicher HB, Kofoed-Hansen B, Jacobsen N (2003). Insecticidal activity of the enantiomers of fipronil. Pest Manag Sci.

[92] Olsvik PA, Williams TD, Tung HS, Mirbahai L, Sanden M, Skjaerven KH, Ellingsen S (2014). Impacts of TCDD and MeHg on DNA methylation in zebrafish (*Danio rerio*) across two generations. Comp Biochem Physiol C: Toxicol Pharmacol.

[93] Aluru N, Kuo E, Helfrich LW, Karchner SI, Linney EA, Pais JE, Franks DG (2015). Developmental exposure to 2,3,7,8-tetrachlorodibenzo-*p*-dioxin alters DNA methyltransferase (dnmt) expression in zebrafish (*Danio rerio*). Toxicol Appl Pharmacol.

[94] Carney SA, Chen J, Burns CG, Xiong KM, Peterson RE, Heideman W (2006). Aryl hydrocarbon receptor activation produces heart-specific transcriptional and toxic responses in developing zebrafish. Mol Pharmacol.

[95] Carney SA, Prasch AL, Heideman W, Peterson RE (2006). Understanding dioxin developmental toxicity using the zebrafish model. Birth Defects Res A Clin Mol Teratol.

[96] Baker TR, King-Heiden TC, Peterson RE, Heideman W (2014). Dioxin induction of transgenerational inheritance of disease in zebrafish. Mol Cell Endocrinol.

[97] Baker TR, Peterson RE, Heideman W (2014). Using zebrafish as a model system for studying the transgenerational effects of dioxin. Toxicol Sci.

[98] Sanchez OF, Lee J, Yu King Hing N, Kim SE, Freeman JL, Yuan C (2017). Lead (Pb) exposure reduces global DNA methylation level by non-competitive inhibition and alteration of dnmt expression. Metallomics.

[99] Knecht AL, Truong L, Marvel SW, Reif DM, Garcia A, Lu C, Simonich MT, Teeguarden JG, Tanguay RL (2017). Transgenerational inheritance of neurobehavioral and physiological deficits from developmental exposure to benzo[*a*]pyrene in zebrafish. Toxicol Appl Pharmacol.

[100] Takata Y, Huang Y, Komoto J, Yamada T, Konishi K, Ogawa H, Gomi T, Fujioka M, Takusagawa F (2003). Catalytic mechanism of glycine *N*-methyltransferase. Biochemistry.

[101] Rowling MJ, McMullen MH, Schalinske KL (2002). Vitamin A and its derivatives induce hepatic glycine *N*-methyltransferase and hypomethylation of DNA in rats. J Nutr.

[102] Luka Z, Capdevila A, Mato JM, Wagner C (2006). A glycine *N*-methyltransferase knockout mouse model for humans with deficiency of this enzyme. Transgenic Res.

[103] Dorts J, Falisse E, Schoofs E, Flamion E, Kestemont P, Silvestre F (2016). DNA methyltransferases and stress-related genes expression in zebrafish larvae after exposure to heat and copper during reprogramming of DNA methylation. Sci Rep.

[104] Klosin A, Lehner B (2016). Mechanisms, timescales and principles of trans-generational epigenetic inheritance in animals. Curr Opin Genet Dev.

[105] Carvan MJ, Kalluvila TA, Klingler RH, Larson JK, Pickens M, Mora-Zamorano FX, Connaughton VP, Sadler-Riggleman I, Beck D, Skinner MK (2017). Mercury-induced epigenetic transgenerational inheritance of abnormal neurobehavior is correlated with sperm epimutations in zebrafish. PLoS ONE.

[106] Stresemann C, Lyko F (2008). Modes of action of the DNA methyltransferase inhibitors azacytidine and decitabine. Int J Cancer.

[107] Kamstra JH, Løken M, Aleström P, Legler J (2015). Dynamics of DNA hydroxymethylation in zebrafish. Zebrafish.

[108] Johns LE, Cooper GS, Galizia A, Meeker JD (2015). Exposure assessment issues in epidemiology studies of phthalates. Environ Int.

[109] Kamstra JH, Sales LB, Aleström P, Legler J (2017). Differential DNA methylation at conserved non-genic elements and evidence for transgenerational inheritance following developmental exposure to mono(2-ethylhexyl) phthalate and 5-azacytidine in zebrafish. Epigenet Chromatin.

[110] Hiller M, Agarwal S, Notwell JH, Parikh R, Guturu H, Wenger AM, Bejerano G (2013). Computational methods to detect conserved non-genic elements in phylogenetically isolated genomes: application to zebrafish. Nucleic Acids Res.

[111] Soares AR, Pereira PM, Ferreira V, Reverendo M, Simões J, Bezerra AR, Moura GR, Santos MA (2012). Ethanol exposure induces upregulation of specific microRNAs in zebrafish embryos. Toxicol Sci.

[112] Lau C, Anitole K, Hodes C, Lai D, Pfahles-Hutchens A, Seed J (2007). Perfluoroalkyl acids: a review of monitoring and toxicological findings. Toxicol Sci.

[113] Zhang L, Li Y, Zeng H, Wei J, Wan Y, Chen J, Xu S (2011). MicroRNA expression changes during zebrafish development induced by perfluorooctane sulfonate. J Appl Toxicol.

[114] Ostrup O, Reiner AH, Aleström P, Collas P (2014). The specific alteration of histone methylation profiles by DZNep during early zebrafish development. Biochim Biophys Acta.

[115] Tan J, Yang X, Zhuang L, Jiang X, Chen W, Lee PL, Karuturi RK, Tan PB, Liu ET, Yu Q (2007). Pharmacologic disruption of polycomb-repressive complex 2-mediated gene repression selectively induces apoptosis in cancer cells. Genes Dev.

[116] Dünzinger U, Haaf T, Zechner U (2007). Conserved synteny of mammalian imprinted genes in chicken, frog, and fish genomes. Cytogenet Genome Res.

[117] Zhuang Z, Wu D, Zhang S, Pang Q, Wang C, Wan R (2006). G-banding patterns of the chromosomes of tonguefish *Cynoglossus semilaevis* Gunther, 1873. J Appl Ichthyol.

[118] Chen S, Tian Y, Yang J, Shao C, Ji X, Zhai J, Liao X, Zhuang Z, Su P, Xu JY, Sha ZX, Wu PF, Wang N (2009). Artificial gynogenesis and sex determination in half-smooth tongue sole (*Cynoglossus semilaevis*). Mar Biotechnol (NY).

[119] Chen S, Zhang G, Shao C, Huang Q, Liu G, Zhang P, Song W, An N, Chalopin D, Volff JN, Hong Y, Li Q, Sha Z, Zhou H, Xie M, Yu Q, Liu Y, Xiang H, Wang N, Wu K, Yang C, Zhou Q, Liao X, Yang L, Hu Q, Zhang J, Meng L, Jin L, Tian Y, Lian J, Yang J, Miao G, Liu S, Liang Z, Yan F, Li Y, Sun B, Zhang H, Zhang J, Zhu Y, Du M, Zhao Y, Schartl M, Tang Q, Wang J (2014). Whole-genome sequence of a flatfish provides insights into ZW sex chromosome evolution and adaptation to a benthic lifestyle. Nat Genet.

[120] Shao C, Li Q, Chen S, Zhang P, Lian J, Hu Q, Sun B, Jin L, Liu S, Wang Z, Zhao H, Jin Z, Liang Z, Li Y, Zheng Q, Zhang Y, Wang J, Zhang G (2014). Epigenetic modification and inheritance in sexual reversal of fish. Genome Res.

[121] Tong SK, Hsu HJ, Chung BC (2010). Zebrafish monosex population reveals female dominance in sex determination and earliest events of gonad differentiation. Dev Biol.

[122] Lombó M, Fernández-Díez C, González-Rojo S, Navarro C, Robles V, Herráez MP (2015). Transgenerational inheritance of heart disorders caused by paternal bisphenol A exposure. Environ Pollut.

[123] Chen J, Xiao Y, Gai Z, Li R, Zhu Z, Bai C, Tanguay RL, Xu X, Huang C, Dong Q (2015). Reproductive toxicity of low level bisphenol A exposures in a two-generation zebrafish assay: evidence of male-specific effects. Aquat Toxicol.

[124] Wu SF, Zhang H, Cairns BR (2011). Genes for embryo development are packaged in blocks of multivalent chromatin in zebrafish sperm. Genome Res.

[125] Lindeman LC, Andersen IS, Reiner AH, Li N, Aanes H, Østrup O, Winata C, Mathavan S, Müller F, Aleström P, Collas P (2011). Prepatterning of developmental gene expression by modified histones before zygotic genome activation. Dev Cell.

[126] Vastenhouw NL, Zhang Y, Woods IG, Imam F, Regev A, Liu XS, Rinn J, Schier AF (2010). Chromatin signature of embryonic pluripotency is established during genome activation. Nature.

[127] Gapp K, Jawaid A, Sarkies P, Bohacek J, Pelczar P, Prados J, Farinelli L, Miska E, Mansuy IM (2014). Implication of sperm RNAs in transgenerational inheritance of the effects of early trauma in mice. Nat Neurosci.

[128] Yan W (2014). Potential roles of noncoding RNAs in environmental epigenetic transgenerational inheritance. Mol Cell Endocrinol.

[129] van de Werken C, van der Heijden GW, Eleveld C, Teeuwssen M, Albert M, Baarends WM, Laven JS, Peters AH, Baart EB (2014). Paternal heterochromatin formation in human embryos is H3K9/HP1 directed and primed by sperm-derived histone modifications. Nat Commun.

